# A Second Life for Seafood Waste: Therapeutical Promises of Polyhydroxynapthoquinones Extracted from Sea Urchin by-Products

**DOI:** 10.3390/antiox12091730

**Published:** 2023-09-07

**Authors:** Luca Melotti, Andrea Venerando, Giulia Zivelonghi, Anna Carolo, Stefania Marzorati, Giordana Martinelli, Michela Sugni, Lisa Maccatrozzo, Marco Patruno

**Affiliations:** 1 Department of Comparative Biomedicine and Food Science, University of Padua, Viale dell’Università 16, 35020 Padova, Italy; luca.melotti@unipd.it (L.M.); giulia.zivelonghi@studenti.unipd.it (G.Z.); anna.carolo@unipd.it (A.C.); lisa.maccatrozzo@unipd.it (L.M.); marco.pat@unipd.it (M.P.); 2 Department of Agricultural, Food, Environmental and Animal Sciences, University of Udine, Via delle Scienze 206, 33100 Udine, Italy; 3 Department of Environmental Science and Policy, University of Milan, Via Celoria 2, 20133 Milan, Italy; stefania.marzorati@unimi.it (S.M.); giordana.martinelli@unimi.it (G.M.); michela.sugni@unimi.it (M.S.)

**Keywords:** polyhydroxynaphtoquinones, spinochromes, sea urchin, antioxidant, marine food waste, zero-waste approach

## Abstract

Coping with a zero-waste, more sustainable economy represents the biggest challenge for food market nowadays. We have previously demonstrated that by applying smart multidisciplinary waste management strategies to purple sea urchin (*Paracentrotus lividus*) food waste, it is possible to obtain both a high biocompatible collagen to produce novel skin substitutes and potent antioxidant pigments, namely polyhydroxynapthoquinones (PHNQs). Herein, we have analyzed the biological activities of the PHNQs extract, composed of Spinochrome A and B, on human skin fibroblast cells to explore their future applicability in the treatment of non-healing skin wounds with the objective of overcoming the excessive oxidative stress that hinders wound tissue regeneration. Our results clearly demonstrate that the antioxidant activity of PHNQs is not restricted to their ability to scavenge reactive oxygen species; rather, it can be traced back to an upregulating effect on the expression of superoxide dismutase 1, one of the major components of the endogenous antioxidant enzymes defense system. In addition, the PHNQs extract, in combination with Antimycin A, displayed a synergistic pro-apoptotic effect, envisaging its possible employment against chemoresistance in cancer treatments. Overall, this study highlights the validity of a zero-waste approach in the seafood chain to obtain high-value products, which, in turn, may be exploited for different biomedical applications.

## 1. Introduction

The skin, as the largest organ of human body, acts as the first line of defense against the external environment, preventing dehydration and defending against harmful stimuli and microorganisms. Its protective role is achieved not only via the physical barrier conferred by the tegument, but also via a complex and finely tuned system involving both inflammatory and immunological processes [1]. The integrity of the skin barrier is mandatory so that it can fulfill its multifunctional roles, and whenever injuries or specific pathological conditions interrupt the physical continuity of functional cutaneous tissue by creating a skin lesion, tissue regeneration and repair processes normally occur. Skin wound healing is a complex multi-phase process, in which different stages, namely, hemostasis, inflammation, proliferation, and remodeling, take place [2]. Several biological components, such as different cell subtypes, growth factors, cytokines, and the extracellular matrix (ECM), among others, participate and tightly regulate the wound healing process [3]. In the cellular environment, reactive oxygen species (ROS), oxygen-derived molecules (including radicals) and unstable oxygen-containing molecules produced mainly by nicotinamide adenine dinucleotide phosphate (NADPH) oxidase and mitochondria respiratory chain [4], deserve particular attention, as they are essential for the stimulation of the wound healing process. Indeed, low levels of ROS drive lymphocytes recruitment at the wound site, mediate vasoconstriction, activate platelets and thrombin, and promote wound debridement along with the clearance of apoptotic/necrotic cells throughout tissue remodeling. In addition, ROS play a fundamental role in innate immunity and pathogen defense [5,6]. However, only finely balanced ROS production and modulation by the endogenous antioxidant enzymes system (i.e., catalases, superoxide dismutases, and glutathione peroxidases) critically determines the effective healing outcome. In fact, elevated and sustained ROS release generated in particular cases by wounded tissues and/or by the bacterial colonization of wounds results in the degradation of ECM proteins and impairment of dermal cell functioning [5]. In addition, the excessive oxidative stress induces strong inflammatory reactions and hinders wound tissue regeneration, eventually leading to chronic non-healing wounds [5,6]. A wound is defined as chronic when (i) it does not heal in the orderly stages of the healing process, (ii) it does not heal within three months, or (iii) it is 40–50% unhealed after four weeks of treatment [7].

Interestingly, it has been proposed that the regulation of redox balance in the wound microenvironment by the modulation of ROS level, either indirectly through the enhancement of the endogenous antioxidant enzymes system or directly using ROS-scavenging agents, may represent a valuable wound healing strategy to treat ‘hard-to-heal’ ulcers. Several compounds of natural origin that exhibit antioxidant capacity, have been proposed and tested for their effect on the healing process, and the interest in using antioxidant compounds for wound treatment is growing [8].

Recently, in an attempt to apply a zero-waste approach to the seafood market and unlock the potential of marine resources, we have demonstrated the feasibility of obtaining highly valuable products by recycling the food waste of the Mediterranean purple sea urchin (*Paracentrotus lividus*) [9,10,11,12,13]. Indeed, after the removal of the edible part (i.e., the gonads) and the extraction of native collagen from the peristomial membrane of the sea urchin (which represents only a small proportion of the food waste) to produce biocompatible biomaterials for skin regeneration, the remaining part (i.e., the test and the spines) was subjected to a solvent-based extraction protocol, yielding Spinochrome A and B polyhydroxynapthoquinones (PHNQs) [12]. Spinochromes belong to a class of small polyphenols present with variable hydroxylated quinonoid chemical structures. They have been identified in different sea urchin species [14,15], in which they are present in several tissues, including the test (shell), spines, gonads, coelomic fluid, and eggs [16]. These marine PHNQs exhibit a wide range of biological activities, and they are of particular interest for pharmaceutical applications [16,17]. As an example, Echinochrome A, the most researched molecule of this family, is the active component of Histochrome™ [18], an antioxidant drug approved in Russia to treat ischemia/reperfusion injury [19,20]. In fact, the PHNQs’ chemical structures make them particularly suitable to scavenge ROS, pointing out their potential use as antioxidants and/or immune modulators in several medical applications [19,21,22].

Herein, the antioxidant activity of Spinochrome A and B previously obtained from *P. lividus* food waste and by-products was evaluated on human skin fibroblasts. The principal aim of the present work was to assess the safety and biological potential of these molecules in order to pave the way for the development of smart skin substitutes, which, originating entirely from sea urchin food waste and by-products to obtain collagen and PHNQs, might support and stimulate the wound healing process.

## 2. Materials and Methods

### 2.1. Extraction of Polyhydroxynaphthoquinones (PHNQs)

Purple sea urchin (*Paracentrotus lividus*) waste used for the isolation of polyhydroxynaphthoquinones (PHNQs) was provided by restaurants in Milan (Italy) after gonad removal. The detailed extraction procedure and characterization of PHNQs are reported in a previous work by Marzorati et al. [12]. Briefly, frozen (−20 °C) sea urchin residues were firstly lyophilized and ground using a Pulverisette 11 knife mill (Fritsch, Milan, Italy), at 10,000 rpm for 20 s in liquid nitrogen to avoid powder heating. The lyophilized powder was firstly processed for supercritical fluid extraction (sc-CO_2_) to yield carotenoids and fatty acids fractions. Then, PHNQs were isolated from the residual biomass by means of formic acid decomposition of the carbonate rich matrix, followed by ethyl acetate counter-extraction of the filtered aqueous phase. The PHNQs-enriched extract was dried from the organic phase using a rotary evaporator at 37 °C under vacuum and stored at −20 °C in dark tubes. The PHNQs content was then characterized and analyzed by ultrahigh performance liquid chromatography-photodiode array-electrospray ionization-high resolution mass spectrometry (UPLC-PDA-ESI-HRMS), as described in [12], confirming the presence of Spinochrome A (67.7 ± 5.6%) and Spinochrome B (32.3 ± 2.5%) with a final PHNQs yield of 0.07 ± 0.01% (*w*/*w*) on starting dry biomass.

### 2.2. Cell Cultures and PHNQs Treatments

Normal human dermal fibroblasts (NHDF) were grown in Dulbecco’s Modified Essential Medium (DMEM; Sigma Merck, Darmstadt, Germany) high glucose (4.5 g L^−1^), supplemented with 20% (*w*/*v*) fetal bovine serum, 2 mM L-glutamine, 100 U mL^−1^ penicillin, and 100 mg mL^−1^ streptomycin (Sigma Merck, Darmstadt, Germany). Cells were cultured in a humidified 5% (*v*/*v*) CO_2_ atmosphere at 37 °C. Cell culture medium was changed every 2–3 days, and cells were used at passage 5–10. For the experiments, the cells were seeded in multiwell plates and grown to sub-confluence (80%) before the PHNQs treatment.

The lyophilized PHNQs extract was reconstituted in Hybri-Max sterile-filtered dimethyl sulfoxide (DMSO; Sigma Merck, Darmstadt, Germany) immediately before its use (stock solution, 10 mg mL^−1^, kept in the dark to avoid any possible photodegradation) and dissolved in culture medium. NHDF cells were incubated with the indicated concentrations of PHNQs extract for 24 h (see below). The final concentration of DMSO in culture medium never exceeded 1% (*v*/*v*).

### 2.3. Cell Viability Assay

The effect of PHNQs on cell viability and cytoprotection against oxidative stress was evaluated using the 3-(4,5-dimethylthiazol-2-yl)-3,5-diphenyltriazolium bromide (MTT) assay (Sigma Merck, Darmstadt, Germany). Briefly, NHDF cells were seeded at a density of 10^4^ cells/well in a 96-well microplate and incubated with increasing concentrations of PHNQs (1–100 μg mL^−1^) for 24 h. When indicated, after the 24 h pre-treatment with PHNQs extract, cell medium was replaced with fresh cell medium containing 5 µM Antimycin A (AMA, Thermo Scientific Chemicals, Milan, Italy), and cells were incubated for additional 2 h. Finally, 10 µL of MTT solution (5 mg mL^−1^ in phosphate-buffered saline solution) were added to each well and incubated for 1 h in the dark at 37 °C and 5% CO_2_. The incubation was stopped by the addition of 20 µL of lysis solution containing 20% (*w*/*v*) sodium dodecyl sulfate and 50% (*v*/*v*) dimethylformamide at pH 4.7. Plates were read for OD at λ = 590 nm using a Victor™ X4 2030 multilabel plate reader (PerkinElmer, Waltham, MA, USA). Cells exposed to 1% DMSO were used as control. The experiment was repeated at least three times.

### 2.4. Fluorescent Dye-Based Oxidative Stress Protection

ROS levels in NHDF cells exposed to an oxidative environment were assessed using the fluorescent probe 5-chloromethyl-2′,7′-dichlorohydrofluorescein diacetate (CM-H2DCFDA; Molecular Probes, Thermo Fisher Scientific, Waltham, MA, USA) while following the manufacturer’s instructions with some modifications. Cells were seeded in a 96-well microplate at a density of 10^4^ cells/well; after 24 h growth in complete medium, the cells were treated with 1–10 µg mL^−1^ PHNQs for 24 h. The tested concentrations of PHNQs were selected basing on cell viability results. Then, NHDF cells were rinsed using 10 mM glucose in phosphate-buffered saline (PBS). Thereafter, 10 µM CM-H2DCFDA dye was added to each well and incubated for 45 min in the dark at 37 °C. Afterwards, cells were washed once with 10 mM glucose in PBS, and finally, they were challenged with 5 µM AMA in the same buffer. An increase in fluorescence was estimated by means of a plate reader (Victor™ X4 2030 multilabel plate reader) at λ = 485 nm (excitation) and λ = 535 nm (emission) for 2 h, with readings every 2 min. Negative controls (cells incubated without the probe and any treatment to detect autofluorescence, and only the probe as a baseline value) and positive controls (cells treated with AMA only) were included in the assay. The experiment was repeated at least three times.

### 2.5. Estimation of Mitochondrial Membrane Potential Preservation

The membrane potential of mitochondria (ΔΨ_M_) in NHDF cells was evaluated using 5,50,6,60-tetrachloro-1,10,3,30-tetraethylbenzimidazolcarbocyanine iodide (JC-1) dye (Invitrogen, Thermo Fisher Scientific, Waltham, MA, USA) while following the manufacturer’s instructions. This dye is a fluorescent cationic probe that is able to selectively accumulate into mitochondria following the electrochemical gradient. Its emission shifts from red to green as the mitochondrial membrane potential decreases [23].

In our experimental model, NHDF cells were seeded at a density of 10^4^ cells/well in 96-well microplates. After 24 h at 37 °C and 5% CO_2_, the cells were exposed to different concentrations of PHNQs (1–10 µg mL^−1^) for 24 h. Then, the cells were rinsed with 10 mM glucose in PBS and challenged with 5 µM AMA diluted in DMEM high glucose without phenol red for 2 h. At the end of the incubation period, the cells were washed once and loaded with 15 µM JC-1 dye for 25 min at 37 °C protected from light. Afterwards, the samples were evaluated using a Victor™ X4 2030 multilabel plate reader for the presence of green monomers, λ = 485 nm (excitation)/535 nm (emission), and red aggregates, λ = 485 nm (excitation)/590 nm (emission).

### 2.6. Protein Extraction and Western Blot Analysis

For proteins extraction, NHDF cells were seeded (20 × 10^4^ cells/well) 24 h before the indicated treatments on 6-well plates. After each treatment, the cells were washed twice with ice-cold PBS, scraped, and lysed in a buffer containing Tris-HCl 50 mM pH 7.5, NaCl 150 mM, and NP40 1% (*v*/*v*). cOmplete™ proteases (Roche, Basel, Switzerland) and phosphatases (Sigma-Aldrich, Milan, Italy) inhibitor cocktails were added to the lysis buffer. Protein concentration was determined by the common Bradford spectrophotometric method that relies on the binding of Coomassie Blue G250 dye to proteins with a maximum absorbance at λ = 595 nm. Equivalent amounts of denatured proteins from each sample (30 μg) were loaded and separated by Tris-Glycine SDS-PAGE. Then, proteins were transferred onto PVDF membranes (Immobilon-P; Millipore, Darmstadt, Germany) using the semi-dry Biometra Fastblot apparatus (Analytik Jena GmbH+Co, Jena, Germany). The membranes were incubated overnight at 4 °C in primary antibodies diluted in 1% (*w*/*v*) bovine serum albumin in Tris-Buffered Saline (TBS) containing 0.1% (*w*/*v*) Tween 20. Primary antibodies were rabbit polyclonal anti-Bcl-2 (sc-492, 1:1000; Santa Cruz Biotechnology, Dallas, TX, USA), rabbit polyclonal anti-Glutathione synthetase (GSS) (A14535, 1:1000; ABclonal, Düsseldorf, Germany), rabbit polyclonal anti-Catalase (A11777, 1:1000; ABclonal, Düsseldorf, Germany), and rabbit polyclonal anti-Superoxide dismutase 1 (SOD1) (A0274, 1:1000; ABclonal, Düsseldorf, Germany). Chemiluminescence signals of the HRP-conjugated secondary antibodies were obtained using the iBright image station (Thermo Fisher Scientific, Milan, Italy) and quantified by ImageJ software. Loading control was provided by mouse monoclonal anti-β-actin antibody (A2228, 1:3000; Sigma Merck, Darmstadt, Germany).

### 2.7. Statistical Analysis

Statistical analysis was performed using GraphPad Prism software version 8, 2019 (GraphPad Software, San Diego, CA, USA). All values are expressed as mean ± standard error of the mean (SEM) of at least 3 replicates. A comparison of more than 2 groups was made via one-way ANOVA using Bonferroni’s ‘post hoc’ test. Differences were considered statistically significant at values of *p* < 0.05.

## 3. Results and Discussion

**Figure 1:**
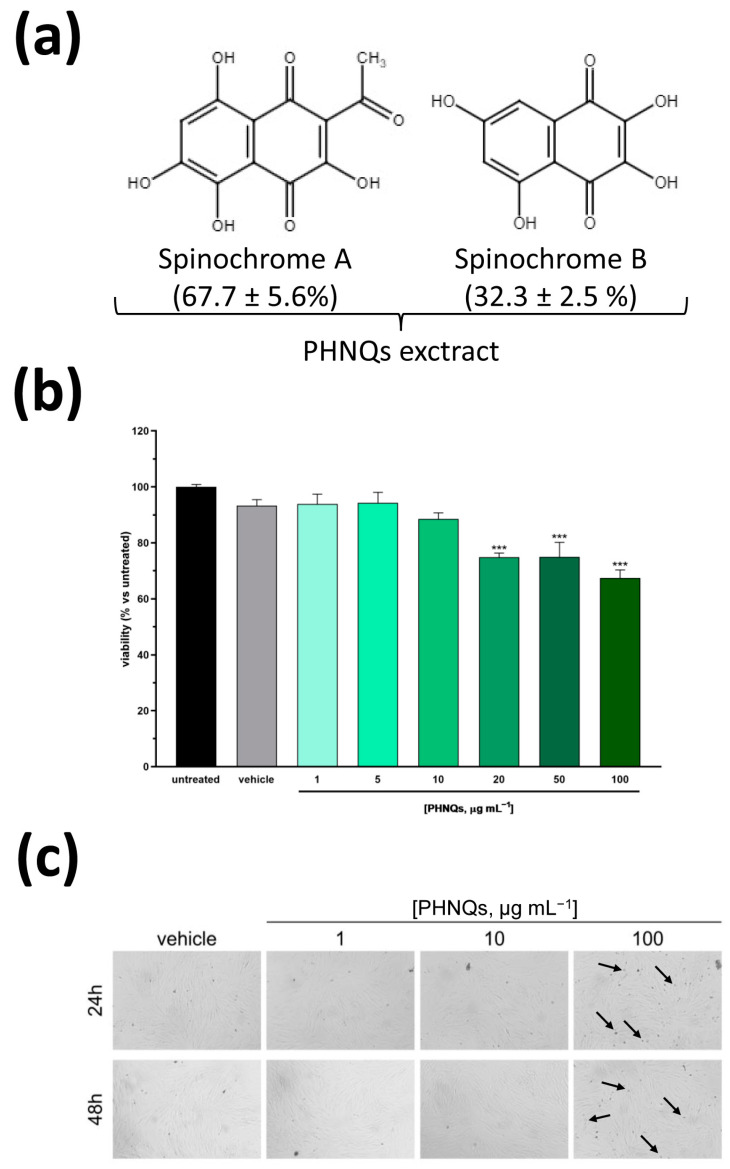
Cytotoxicity evaluation of Spinochrome A and B in human skin cells. (**a**) Chemical structure of the polyhydroxynapthoquinones (PHNQs) extracted from *P. lividus* food waste used in this study. The percentage of each Spinochrome composing the PHNQs extract is reported. Spinochrome A (2-Acetyl-3,5,6,8-tetrahydroxy-1,4-naphthoquinone), MW = 264.19 g mol^−1^; Spinochrome B (2,3,5,7-tetrahydroxy-1,4-naphthoquinone), MW = 222.15 g mol^−1^. (**b**) Normal human dermal fibroblasts (NHDF) viability after exposure to different concentration of Spinochrome A and B for 24 h was evaluated by MTT assay. Viability is expressed as % of untreated cells. Data are expressed as mean ± SEM of three independent experiments; *** *p* < 0.001 compared to untreated samples (one-way ANOVA using Bonferroni’s post-test). (**c**) Representative microphotographs of NHDF exposed to the indicated concentrations of PHNQs extract for 24 (upper panel) and 48 (lower panel) hours. No evident signs of toxicity are present for any of the concentrations tested apart from a limited formation of budding vesicles (indicated by arrows) only visible in the samples treated with 100 μg mL^−1^ PHNQs at both timepoints.

It is generally recognized that food waste causes substantial negative environmental, social, and economic impacts. Notwithstanding, it might represent an extraordinary reservoir of high-value products that, in turn, can contribute to a more sustainable circular economy. In this respect, purple sea urchin (*Paracentrotus lividus*) food waste, which represents the majority of this popular delicacy in the Mediterranean area as well as in the Far East, has been previously exploited as an eco-friendly source of a particular type of collagen in its native fibrillar and glycosaminoglycans (GAG)-decorated conformation, particularly suitable to produce advanced biomimetic biomaterials to treat skin wounds [11,13]. In fact, echinoderms, including sea urchins, possess a particular connective tissue (from which collagen can be easily extracted) that is called Mutable Collagenous Tissue (MCT). MCT presents with peculiar features and the unique ability to undergo rapid changes in its mechanical properties, which are considered at the basis of the remarkable regenerative abilities of this marine organism [24,25,26,27]. However, this highly biomimetic collagen, which is obtained from a limited portion of the sea urchin waste (i.e., the peristomial membrane), represents only one of the products obtainable by the application of smart recycle strategies. Indeed, recently, it has been reported that the remaining carbonate-rich test and spines of sea urchin food waste subjected to a multistep extraction procedure, efficiently yield polyhydroxylatednaphtoquinone (PHNQ) molecules, namely Spinochrome A and Spinochrome B (Figure 1a) [12]. Notably, the PHNQs extract, composed of Spinochrome A (67.7 ± 5.6%) and Spinochrome B (32.3 ± 2.5%), possess a high radical scavenging activity in terms of Trolox^®^ equivalents as demonstrated by ABTS in in vitro extracellular assay [12]. This result prompted us to evaluate the biological effects of PHNQs extract in a more complex scenario, such as in skin cells, in order to pave the way for its potential application in improving the sea urchin collagen-based skin substitute [13] to treat ‘hard-to-heal’ wounds.

### 3.1. Spinochrome A and B Do Not Show Cytotoxic Effects at Low Concentrations

Considering that several naphtoquinones are classified as harmful or very toxic [28], we firstly assessed the cytotoxicity of PHNQs extract. Normal human dermal skin fibroblasts (NHDF) were incubated with increasing concentrations of sea urchin extract containing Spinochromes A and B for 24 h. Cell viability was measured by the MTT-based assay, which relies on the color change of MTT by mitochondrial succinate dehydrogenase. As depicted in Figure 1b, up to 10 μg mL^−1^ PHNQs extract did not affect cell viability substantially. Conversely, treatment with higher concentrations evidenced a dose-dependent, albeit limited, cytotoxicity compared to vehicle alone (DMSO). Indeed, as reported in Figure 1c, the morphological analysis of NHDF cells treated for 24 and 48 h with PHNQs extract substantiated what was evidenced by the MTT assay, revealing the presence of budding vesicles (probably apoptotic bodies) only in cells exposed to the highest concentration tested. On the basis of these results, in the following experiments, PHNQs extract was used in the concentration range (1–10 μg mL^−1^) that did not show any signs of cell toxicity.

### 3.2. Low Concentrations of Spinochrome A and B Exhibit a Cytoprotective Effect in a Redox Environment Preventing the Impairment of the Mithocondrial Membrane

Every day, living cells are subjected to the harmful effects of either exogenously or endogenously produced highly reactive oxidizing molecules such as ROS. After cutaneous injury, ROS are produced in high amounts at the wound site as a natural defense against invading microorganisms [29]. However, although redox signaling plays a fundamental role in regulating every step of normal wound healing, an imbalance between ROS exposure and the endogenous antioxidant defense system may lead to excessive and uncontrolled intracellular oxidative stress. This unbalanced environment sustains and deregulates inflammation processes, which in turn reduce or even block the healing process, as is the case of chronic non-healing wounds [5].

Therefore, to mimic the excessive oxidative stress that may occur at the wound site and that would be deleterious for wound healing, in the present work, NHDF cells were exposed to the antibiotic Antimycin A (AMA), a well-known inhibitor of the electron transport chain at mitochondrial complex III [30,31]. AMA inhibits the oxidation of ubiquinol in the electron transport chain of oxidative phosphorylation by binding to the Qi site of cytochrome c reductase, disrupting the formation of the proton gradient across the inner mitochondrial membrane. As a result, protons are unable to flow through the ATP synthase complex, and the production of ATP is therefore inhibited. Furthermore, treatment with AMA results in an elevated production and accumulation of ROS, which causes the peroxidation of mitochondrial DNA, lipids, and proteins. Thus, to investigate the intracellular antioxidant potential of PHNQs extract, we monitored the changes in ROS release after AMA challenge using the fluorescence probe acetic 5-(chloromethyl)-2-(3,6-diacetoxy-2,7-dichloro-9H-xanthen-9-yl)benzoic anhydride (CM-H2DCFDA).

**Figure 2:**
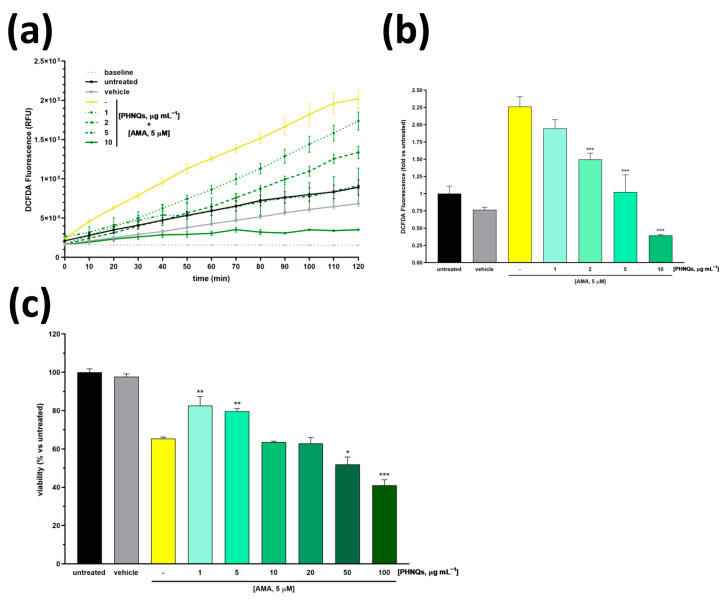
Scavenging activity and cytoprotection of PHNQs against oxidative stress. (**a**) Kinetic evaluation of ROS levels in NHDF pre-treated with different concentrations of PHNQs (1–10 µg mL^−1^) for 24 h and then exposed to 5 µM AMA. In (**b**), the ROS levels at the endpoint of 120 min measured in (**a**) are reported as fold increase with respect to the untreated cells. (**c**) Cell viability of NHDF pre-treated with PHNQs (1–10 µg mL^−1^) and exposed to 5 µM AMA for 2 h was assessed using MTT assay. Viability is expressed as % of untreated cells. Means ± SEM of three independent experiments are reported; * *p* < 0.05, ** *p* < 0.01, *** *p* < 0.001 versus AMA-treated samples (yellow column) using one-way ANOVA with Bonferroni’s post-test.

Conversely to H2DCFDA, the oxidation of CM-H2DCFDA yields a fluorescent adduct that is trapped inside the cell, thereby avoiding any leakage of signal in living cells analysis. Interestingly, the pre-treatment of skin fibroblasts with increasing concentrations of the PHNQs extract for 24 h was able to reduce the production of intracellular ROS induced by AMA treatment (Figure 2a). Notably, 5 μg mL^−1^ of PHNQs extract almost completely reverted the signal to that obtained in untreated cells, and the pre-treatment with 10 μg mL^−1^ of extract approached the baseline (Figure 2b).

The protective effect of the PHNQs extract against the AMA treatment was also demonstrated on the metabolic activity of skin fibroblast cells using the MTT assay (Figure 2c). As reported by the authors of [32], AMA significantly reduces the metabolic activity (i.e., viability) of several cell lines. Worthy of note, when skin fibroblasts were pre-incubated with PHNQs extract, the AMA-induced cytotoxicity was partially suppressed. However, it should be noted that if low amounts of PHNQs (up to 5 μg mL^−1^) mitigated the harmfulness of AMA, higher doses would demonstrate a synergic effect resulting in enhanced cytotoxicity (Figure 2c). Overall, these results suggest that low doses of PHNQs extract might protect skin fibroblasts against ROS-induced cell death.

**Figure 3:**
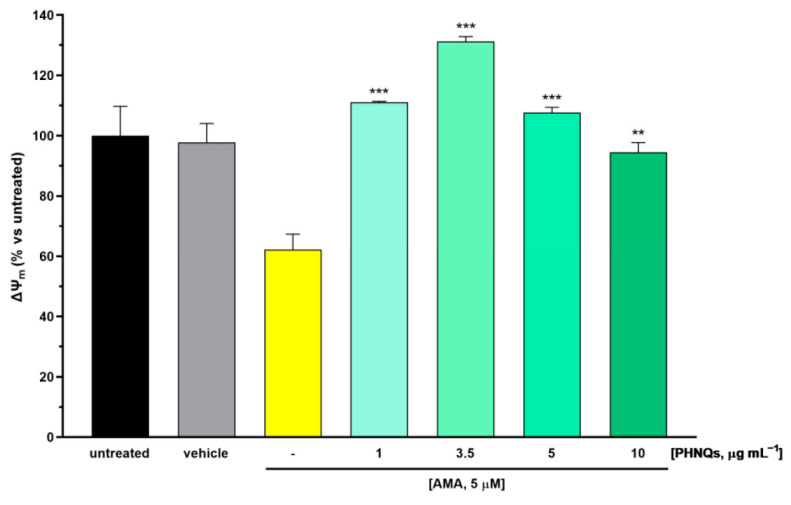
PHNQs effect on AMA-induced mitochondrial membrane depolarization. NHDF were pre-incubated with PHNQs extract (1–10 µg mL^−1^) for 24 h and then challenged with 5 µM AMA for an additional 2 h. Mitochondrial membrane potential (ΔΨ_M_ values expressed as % of untreated cells) were obtained from the ratio of red fluorescence (aggregates) and green fluorescence (monomers) of the potential-dependent JC-1 fluorescent dye, as described in the Material and Methods section. Data are expressed as mean ± SEM of experiments performed in triplicate; ** *p* < 0.01, *** *p* < 0.001 compared to AMA-treated samples (yellow column) using one-way ANOVA with Bonferroni’s post-test.

Increasing ROS cell content due to the AMA-induced blocking of electron transfer at complex III promotes outer mitochondrial membrane permeabilization through the opening of the mitochondrial permeability transition pore, which in turn leads to mitochondrial membrane potential (ΔΨ_M_) collapse and the depolarization of mitochondria [33]. The protective effect of PHNQs extract on ΔΨ_M_ changes in AMA-treated skin cells was measured by JC-1 dye staining. JC-1 fluorescent cationic carbocyanine dye exhibits potential-dependent accumulation in mitochondria [23]. Indeed, in healthy cells where ΔΨ_M_ is high, JC-1 accumulates in mitochondria, forming aggregates with intense red fluorescence. On the contrary, when ΔΨ_M_ drops down, as is the case of AMA-induced depolarization, JC-1 remains in the monomeric form, which presents green fluorescence. As shown in Figure 3, skin fibroblast cells treated with AMA displayed a significant decrease in the ΔΨ_M_ value when compared with untreated samples. Notably, pre-incubation with PHNQs extract preserved ΔΨ_M_ from AMA-induced mitochondrial depolarization. Nevertheless, it should be pointed out that high concentrations of PHNQs extract demonstrated a partial loss of their protective effect.

### 3.3. Exposure to Spinochrome A and B Boosts Antimycin A-Induced Pro-Apoptotic Effect in a Dose-Dependent Manner

**Figure 4:**
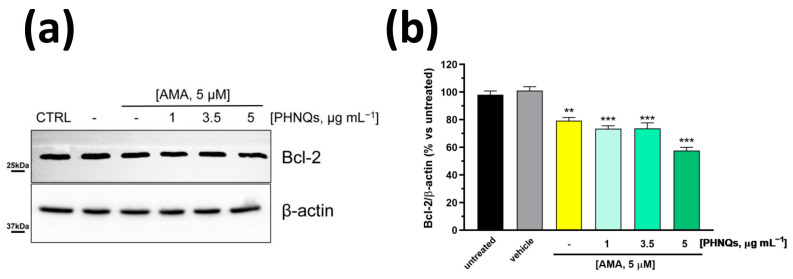
PHNQs extract enhances the downregulation of Bcl-2 anti-apoptotic protein induced by Antimycin A treatment. NHDF were pre-treated for 24 h with PHNQs at different concentrations and then challenged with 5 μM AMA for 2 h. (**a**) Total cell lysate (30 µg proteins/sample) were separated by SDS-PAGE and immunoblotted with antibodies against B-cell lymphoma 2 (Bcl-2) protein and β-actin (loading control). Representative Western blots of three independent experiments are shown. In (**b**), the densitometric analysis of proteins expression reported as % of untreated cells is shown. Data are expressed as mean ± SEM; ** *p* < 0.01, *** *p* < 0.001 compared to untreated cells (one-way ANOVA with Bonferroni’s post-test).

Apoptosis is a highly regulated process of programmed cell death that is activated in response to stress-inducing signals [34]. High ROS concentrations, which can cause or might be derived from the impairment of mitochondrial function and structure, initiate and mediate apoptosis signaling cascade. Further, mitochondrial depolarization that occurs in the early stages of apoptosis is followed by the release of pro-apoptotic factors, such as cytochrome c and AIF (apoptosis-inducing factor) [35]. It is already known that Antimycin A induces apoptosis [36] and that it is able to downregulate B-cell lymphoma 2 (Bcl-2) protein in cancer cells [37,38]. Bcl-2 protein belongs to the group of anti-apoptotic survival Bcl-2 family members [39], and its overexpression is considered a protecting factor from ROS-mediated apoptosis in cells [40]. In the context of PHNQ molecules, it has been reported that Histochrome™, the sodium salt of echinochrome A, significantly upregulates Bcl-2 protein in cardiac progenitor cells, protecting them from oxidative stress-induced apoptosis [19]. For this reason, we investigated whether PHNQs extract might counteract the AMA-induced apoptotic effect by monitoring Bcl-2 protein level. Unexpectedly, the 24 h pre-treatment with PHNQs demonstrated a dose-dependent synergistic effect with AMA by lowering the total amount of Bcl-2 protein in human skin fibroblasts (Figure 4).

### 3.4. Spinochrome A and B Enhance SOD1 Levels in Human Skin Fibroblasts

**Figure 5:**
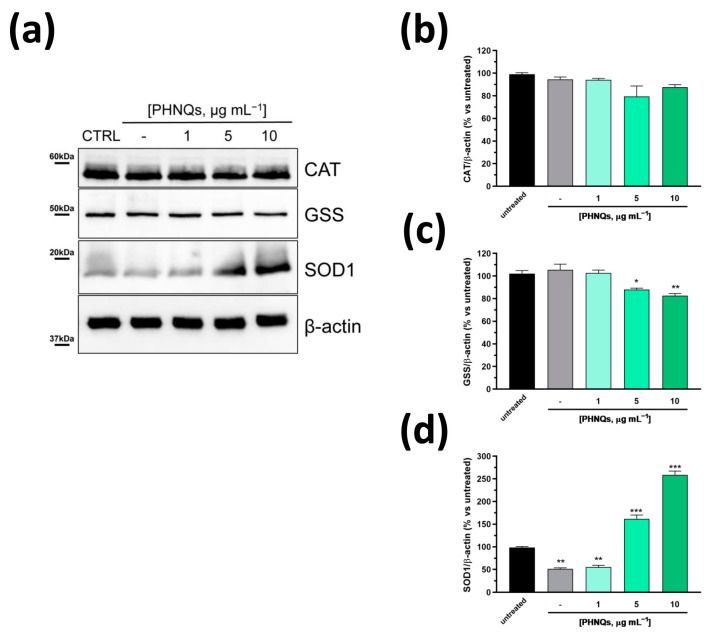
PHNQs influence on key regulators enzymes of oxidative stress. (**a**) Representative Western blot analysis of different concentrations of PHNQs tested on NHDF for 24 h. Total cell lysate (30 µg proteins/sample) was separated by SDS-PAGE and immunoblotted with antibodies against the indicated antioxidant enzymes. β-actin was used as loading control. Densitometric analysis of (**b**) catalase (CAT), (**c**) glutathione synthetase (GSS), and (**d**) superoxide dismutase 1 (SOD1). Protein band intensities were normalized to β-actin and expressed as % of untreated cells. Values are reported as mean ± SEM of three independent experiments; * *p* < 0.05, ** *p* < 0.01, *** *p* < 0.001 compared to untreated samples using one-way ANOVA with Bonferroni’s post-test.

Several studies have demonstrated the reciprocal relationship between ROS and Bcl-2 levels in cells [40]. Generally, ROS decrease correlates with the increase in Bcl-2 protein amount in various cells. However, in our experimental model, PHNQs extract was able to decrease AMA-induced ROS amount (Figure 2a,b) but, conversely, it downregulated Bcl-2 protein level (Figure 4b). Note that Bcl-2 itself does not present any antioxidant activity [41]. On the contrary, within cells, Bcl-2 may indirectly sustain the endogenous antioxidant defense system by increasing the amount and/or activities of antioxidant enzymes [41]. Thus, to investigate the effect of PHNQs on the relationship between Bcl-2 and the major antioxidant defense systems (i.e., glutathione synthetase, catalase and superoxide dismutase 1), NHDF cells were treated with increasing amounts of PHNQs extract from sea urchin waste for 24 h without the following challenge with AMA in order to exclude any influence by the antibiotic. As reported in Figure 5a, PHNQs extract did not affect the amount of catalase (Figure 5b), rather it slightly decreased the glutathione synthetase protein level (Figure 5c). On the contrary, as shown in Figure 5d, the superoxide dismutase 1 (SOD1) protein expression level was greatly upregulated by treatment with PHNQs extract in a dose-dependent manner despite the detrimental effect exhibited by vehicle alone (i.e., DMSO).

## 4. Conclusions

In this study, small polyhydroxynapthoquinones (PHNQs), namely Spinochromes A and B, extracted from sea urchin food waste and characterized in a previous work by our group [12], were successfully tested in human skin dermal fibroblasts for their ability to prevent excessive oxidative stress that may occur at a wound site. Notably, our data demonstrated that the antioxidant potential of Spinochromes A and B is not restricted to free radical and ROS scavenging properties, as previously reported for this class of naturally occurring molecules [16,42]. Indeed, they are able to upregulate superoxide dismutase 1, one of the major component of the endogenous antioxidant enzymes defense system [43]. It should be stressed that, among the antioxidant enzymes, the family of metalloenzymes superoxide dismutases represents the first line of defense in removing ROS excess by converting the superoxide radical anion into molecular oxygen and hydrogen peroxide, which is further eliminated by the other antioxidant enzymes such as catalase, glutathione peroxidases, and peroxiredoxins [44]. Overall, these results further support the future employment of PHNQs in the development of smart antioxidant collagen-based skin substitutes, which are completely made starting from sea urchin food waste, for the treatment of wounds.

Finally, from a different point of view, the synergistic effect demonstrated by PHNQs extract with Antimycin A in downregulating the anti-apoptotic Bcl-2 protein might be instrumental for a possible re-evaluation of Antimycin A in a combined therapy to overcome chemoresistance in cancer. In fact, it was reported that the overexpression of Bcl-2 confers chemoresistance to cancer cells [45]. Therefore, Bcl-2 (as well as other Bcl-2 family members) has become an interesting target for drug development in combination with conventional chemotherapy to sensitize resistant cancer cells [37]. In this respect, Antimycin A has been proposed as an inhibitor of the anti-apoptotic Bcl-2 proteins, but its use was limited due to the high concentrations required to block cancer cells growth in vivo [32]. Interestingly, our results unveil an interesting new biological activity of Spinochrome A and B that would merit a thorough future investigation.

## Data Availability

The data presented in this study are available in the article and from the authors upon request.
